# Zoonotic neglected tropical diseases at the animal-human interface in the Greater Mekong Subregion: Two decades of surveillance in Laos and Cambodia

**DOI:** 10.1371/journal.pntd.0014584

**Published:** 2026-07-30

**Authors:** Stuart D. Blacksell, Michael P. Ward, James R. Young, Russell D. Bush, Peter A. Windsor, Syseng Khounsy, Phouvong Phommachanh, Watthana Theppangna, Jarunee Siengsanan-Lamont, Rebekah Burns, Paul Turner, Jeffrey Gilbert, Lida Kong, Sothyra Tum, Erik A. Karlsson, James V. Conlan, Tom Hughes, Jaison B. Kolenchery, Tamalee Roberts, Matthew T. Robinson, Manivanh Vongsouvath, Paul N. Newton, Mayfong Mayxay, Elizabeth A. Ashley, Nicholas P. J. Day

**Affiliations:** 1 Mahidol Oxford Tropical Medicine Research Unit (MORU), Faculty of Tropical Medicine, Mahidol University, Bangkok, Thailand; 2 Centre for Tropical Medicine and Global Health, Nuffield Department of Medicine, University of Oxford, Oxford, United Kingdom; 3 Sydney School of Veterinary Science, Faculty of Science, The University of Sydney, Camperdown, NSW, Australia; 4 National Animal Health Laboratory, Vientiane, Lao People’s Democratic Republic; 5 Cambodia-Oxford Medical Research Unit, Angkor Hospital for Children, Siem Reap, Cambodia; 6 World Health Organization, Geneva, Switzerland; 7 National Animal Health and Production Research Institute, Phnom Penh, Cambodia; 8 Virology Unit, Institut Pasteur du Cambodge, Phnom Penh, Cambodia; 9 Food Standards Australia New Zealand, Canberra, Australian Capital Territory, Australia; 10 Conservation Medicine, Sungai Buloh, Selangor, Malaysia; 11 Lao-Oxford-Mahosot Hospital-Wellcome Trust Research Unit (LOMWRU), Mahosot Hospital, Vientiane, Lao People’s Democratic Republic; 12 Institute for Research and Education Development, University of Health Sciences, Vientiane, Lao People’s Democratic Republic; 13 Lao One Health University Network (LAOHUN), Vientiane, Lao People’s Democratic Republic; 14 Saw Swee Hock School of Public Health, National University of Singapore (NUS), Singapore, Singapore; Institute of Continuing Medical Education of Ioannina, GREECE

## Abstract

Zoonotic neglected tropical diseases (NTDs) remain a substantial but under-recognised source of human morbidity and economic concern in the Greater Mekong Subregion, particularly in settings characterised by close human–animal interaction. This article represents a narrative synthesis of zoonotic disease research conducted in Laos and Cambodia between 2000 and 2025. The review integrates published literature with findings from long-term surveillance programmes conducted by the authors and collaborating institutions in Laos, with comparative insights from Cambodia, to examine the presence, distribution, diversity, and drivers of zoonotic pathogens at the human–animal interface. Evidence demonstrates the endemic presence of a wide range of parasitic, bacterial, and viral zoonoses, including *Taenia solium*, *Trichinella* spp., *Streptococcus suis*, rickettsial infections, melioidosis, hepatitis E virus, and Japanese encephalitis virus. Some of these pathogens are sustained within smallholder livestock systems, informal slaughter, farming practises, food networks, and wet market environments, where limited diagnostic capacity and fragmented surveillance obscure true disease presence. Surveillance innovations, including abattoir-based sampling, cross-sectoral serological studies, environmental surveillance approaches, and molecular diagnostic tools, have improved pathogen detection but have also highlighted persistent structural and behavioural barriers to control. Socio-cultural practices, occupational exposure, wildlife trade, and economic dependencies reinforce transmission dynamics, indicating that biomedical interventions alone are insufficient. Instead, zoonotic disease persistence reflects the interaction of livestock production systems, environmental conditions, diagnostic limitations, and entrenched human behaviours. This review emphasises the need for integrated One Health approaches that combine strengthened surveillance, improved diagnostics, behavioural interventions, and regional collaboration. Addressing zoonotic NTDs in Laos and Cambodia requires coordinated strategies that account for both biological complexity and socio-economic context to achieve sustainable disease control and improved public health outcomes.

## Author summary

Many infectious diseases that affect people in Southeast Asia originate from animals, particularly in rural communities where livestock are raised close to households and food is often prepared using traditional methods. In Laos and Cambodia, these zoonotic diseases are common but frequently overlooked because they are difficult to diagnose and are not routinely reported.

In this review, we bring together findings from over 20 years of research to show that a wide range of zoonotic infections, caused by parasites, bacteria, and viruses, continue to circulate between animals and humans. These include infections linked to pigs, poultry, wildlife, and environmental exposure. We show that these diseases persist not because they are poorly understood, but because of deeper challenges such as limited laboratory capacity, informal food systems, cultural practices (such as eating raw meat), and economic reliance on small-scale livestock farming.

Our findings highlight that improving human health requires more than medical solutions alone. Effective control will depend on coordinated “One Health” approaches that integrate human, animal, and environmental health, while also addressing the social and economic realities faced by affected communities. Strengthening surveillance, improving diagnostics, and working closely with communities will be essential to reducing the presence of zoonotic diseases in the region.

## Introduction

Zoonotic diseases remain a persistent challenge at the human-animal-environment interface, particularly in low- and middle-income countries where endemic infections often receive less attention than epidemic threats. The World Health Organization (WHO), together with the Food and Agriculture Organization (FAO) and the World Organisation for Animal Health (WOAH), have emphasised that endemic zoonotic diseases are frequently under-detected due to fragmented surveillance, limited diagnostic capacity, and weak coordination between human and animal health sectors [1]. These gaps are especially pronounced in settings characterised by smallholder livestock production, informal slaughter, and close human-animal contact. Many of these infections meet the WHO’s definition of neglected tropical diseases (NTDs) [2], disproportionately affecting poor and rural communities while receiving limited attention from health systems, surveillance programmes, and policy agendas.

The Lao PDR (Laos) and Cambodia are both low- and middle-income countries (LMICs) located in the Greater Mekong Subregion (GMS) of Southeast Asia. Smallholder and backyard livestock production systems are central to rural livelihoods, food security, and cultural practices, but they also create sustained opportunities for the transmission of zoonotic pathogens [3]. Livestock-associated zoonotic risks are influenced by traditional animal husbandry, informal slaughter, inappropriate handling and food preparation practices, including the consumption of raw or fermented meat and fresh animal blood, limited veterinary infrastructure, and porous borders that facilitate transboundary transmission [4]. These structural and behavioural factors intersect with poverty, occupational exposure, and constrained access to healthcare, amplifying the impact of neglected zoonotic diseases on vulnerable populations.

This review synthesises evidence from two decades of collaborative research in Laos and comparative findings from Cambodia to examine the presence, distribution, diversity and drivers of zoonotic diseases at the human-animal interface. Systematic analysis of historical pathogen discovery data has been used to estimate national human pathogen diversity and model discovery dynamics.

This review is framed within the One Health approach as defined by the One Health High‑Level Expert Panel (OHHLEP), an interdisciplinary advisory body to the FAO, WHO, WOAH and UNEP (United Nations Environment Programme), which emphasises the interconnected and interdependent health of humans, animals and ecosystems [5]. The review examines zoonotic disease persistence through four interacting domains: (i) livestock production systems, (ii) environmental and ecological drivers, (iii) diagnostic and surveillance capacity, and (iv) socio-cultural practices that shape human–animal contact. These domains provide the analytical framework for interpreting evidence from two decades of surveillance in Laos and Cambodia. By focussing on why zoonotic NTDs persist despite the availability of practical biomedical tools, this review aims to identify priority gaps, inform integrated One Health surveillance, prevention, and control strategies, and highlight pathogen-specific opportunities for integrated interventions in Laos and Cambodia.

Effective One Health implementation extends beyond the biomedical and veterinary sciences to include social and behavioural sciences, economics, law and policy, environmental sciences, communication, data science, and public policy. These disciplines contribute to understanding human behaviour, evaluating intervention costs, strengthening legal and governance frameworks, improving risk communication, and integrating surveillance across sectors.

For the purposes of this review, we use the term zoonotic diseases in a broad One Health context to include pathogens maintained at the animal–human interface, including classical zoonoses transmitted directly or indirectly from vertebrate animals, foodborne zoonoses, vector-borne zoonoses, and environmentally acquired infections in which animal reservoirs contribute to pathogen maintenance (e.g., melioidosis, Q fever). Where appropriate, we distinguish environmentally acquired sapronotic infections from classical zoonoses.

## Methods

**Fig 1:**
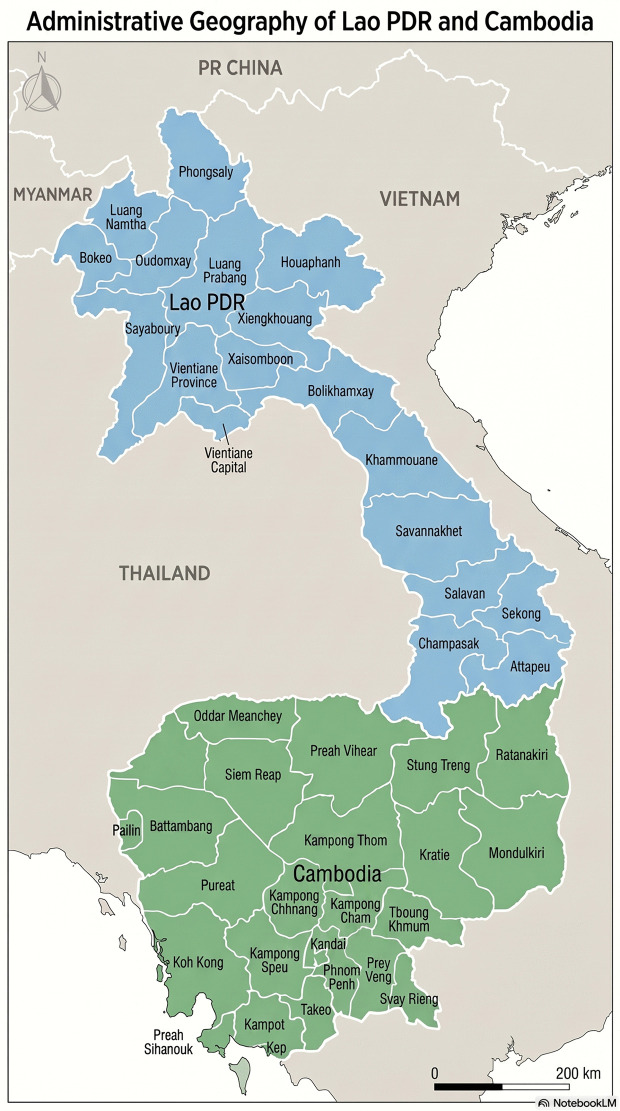
Study region and administrative boundaries of Laos and Cambodia. The map was created in QGIS version 3.44.7-Solothurn using administrative boundary datasets obtained from Natural Earth (https://www.naturalearthdata.com/). Country boundaries were derived from the Natural Earth Admin 0 – Countries dataset and provincial boundaries from the Natural Earth Admin 1 – States, Provinces dataset. Natural Earth data are in the public domain.

This article represents a review of zoonotic disease research conducted in Laos and Cambodia between 2000 and 2025. This review was intended as a narrative synthesis rather than a systematic review. The review was not intended to provide a comprehensive, systematic assessment of all published literature on zoonotic diseases in the region. The review integrates published literature with findings from long-term surveillance programmes conducted by the authors and collaborating institutions in Laos and Cambodia. Relevant studies were identified through targeted searches of PubMed, Web of Science, and institutional project outputs, combined with expert knowledge of long-running surveillance programmes in the region. Search terms included combinations of “Laos”, “Cambodia”, “Greater Mekong Subregion”, “zoonosis”, “zoonotic disease”, “One Health”, “neglected tropical disease”, together with pathogen-specific terms. Only publications in English language were included. Pathogens were included if they had documented zoonotic transmission pathways, evidence of occurrence in Laos or Cambodia, recognised public health significance, or relevance to One Health surveillance and control programmes. A map showing the provinces of Laos and Cambodia described in the studies is presented in Fig 1.

Long-term surveillance data were derived from programmes conducted between 2000 and 2025 through MORU, LOMWRU, DLF, COMRU and collaborating institutions. These included hospital-based febrile illness surveillance, encephalitis surveillance, livestock serosurveys, abattoir-based surveillance, market-based studies, and outbreak investigations across multiple provinces in Laos and Cambodia. Not all surveillance programmes covered all pathogens or geographic regions; rather, the review synthesises evidence generated through complementary surveillance activities over two decades.

## Results

### Foodborne zoonoses

#### *Taenia* spp*.*

Taeniasis and cysticercosis remain important but under-recognised parasitic infections in Laos, with human cysticercosis representing the tissue-invasive consequence of *Taenia solium* infection and a significant cause of epilepsy and neurological disease in endemic areas [6,7]. In the late 2000s, collaborative research between the Lao Department of Livestock and Fisheries (DLF), Murdoch University, Mahidol Oxford Tropical Medicine Research Unit (MORU) and funded by the Australian Centre for International Agricultural Research (ACIAR) confirmed the endemic presence of *Taenia solium* and other taeniid cestodes in pigs, underscoring their ongoing importance as public health and development concerns in Laos. *T. solium* was consistently identified in smallholder pig populations, with prevalence varying significantly across ecological zones [6,8]. These studies revealed that infection risk was closely associated with open defecation, free-roaming pig management, and limited access to veterinary services, all of which remain widespread in rural communities [6,8]. Cross-sectional surveys across four northern provinces estimated human cysticercosis prevalence of 2.2% (95% CI 1.4–3.0) and taeniasis prevalence of 8.4% (95% CI 6.9%–9.9%). Among recovered tapeworms, 94.3% were *T. saginata* and only 5.7% were *T. solium*. In pigs, maximum-likelihood adjusted prevalence was 4.2% for *T. solium* (95% CI 0.5%–7.9%) compared with 55.9% for *T. hydatigena* (95% CI 47.5%–64.3%). These findings support the hypothesis that interspecific competition among taeniids may suppress *T. solium* transmission in this multi-endemic setting [9]. Such ecological interactions may influence transmission dynamics and should be considered when designing parasite control strategies. Key findings are summarised in Tables 1 and 2. Comparative drivers and intervention opportunities are summarised in Table 3.

**Table 1:** Summary of key zoonotic pathogens at the animal–human interface in Laos, including host species, sample sizes, prevalence, location, study period, and primary data sources.

Disease/pathogen	Host(s)	Sample size	Key prevalence/findings	Location	Year(s)	Primary citation(s)
Melioidosis (*Burkholderia pseudomallei*)	Cattle, buffalo, pigs	917 livestock	Cattle 22.8%; buffalo 19.7%; pigs 4.0%; seasonal peak 17.0% (Jan 2020); spatial cluster in Savannakhet (*p* = 0.041)	Nationwide (cluster SW Laos)	2020	[132]
	Hospital patients	Case series	Endemic cause of severe sepsis and pneumonia; high case fatality; underdiagnosed outside referral centres	Nationwide	Ongoing	[127–129]
*Rickettsia*/ *Orientia* spp.	Cattle, pigs, buffalo	821 livestock	3.9% overall; cattle 9.9%, pigs 2.4%, buffalo 0%; southern cluster (*p* = 0.0056)	18 provinces	2022–2023	[78]
	Febrile patients	Prospective cohorts	Major causes of acute undifferentiated fever (scrub typhus, murine typhus)	Vientiane; national	2000s–ongoing	[75–77]
Brucellosis (*Brucella* spp.)	Cattle, buffalo	683	0.2% seropositive (abattoir survey)	6 provinces	2019	[88]
	Goats	1,458	~1.4% seropositive; risk linked to province, breed, age	5 provinces	2016–2018	[86]
Q Fever (*Coxiella burnetii*)	Goats	1,458	4.1% seropositive; province, breed, age ≥3 years significant	5 provinces	2016–2018	[86]
Hepatitis E Virus (HEV)	Pigs	586 (abattoir); 301 (village); 181 PCR	51.2% (abattoir), 15.3% (village); 11.6% RNA positive; genotype 4	Northern Laos	2007–2011	[32,33]
	General population	2,412 individuals	57.9% anti-HEV IgG; age-related increase; geographic heterogeneity	5 provinces	2025	[34]
	Hospital patients	Case series	Low clinical detection despite widespread exposure	Vientiane	2010	[35]
Japanese Encephalitis Virus (JEV)	Pigs	724	74.7% seroprevalence; IgM 2.3% (recent infection)	Northern Laos	2008–2009	[71]
	CNS infection patients	Prospective surveillance	Leading cause of viral encephalitis; major paediatric disease	Nationwide	2003–2020	[54–56]
*Trichinella* spp.	Pigs	728	2.1% infected (*T. spiralis*)	Northern Laos	2004–2010	[18–20]
	Community survey	1,419 individuals	19.1% seropositive (95% CI 17.1–21.1); outbreak (~650 cases, 2005)	Northern Laos	2004–2010	[18–20]
*Taenia solium* & other taeniids	Pigs	590	*T. solium* 4.2%; *T. hydatigena* 55.9%	Northern Laos	~2012	[8,9]
	Dogs	105	Reservoir hosts identified	Northern Laos	~2012	[9]
	Community survey	1,582 individuals	Cysticercosis 2.2%; taeniasis 8.4%; majority *T. saginata*	Northern Laos	~2012	[6,9]
Rabies virus	Dogs, humans	Not specified	Endemic; dog-mediated transmission; substantial underreporting due to limited diagnostics and surveillance	Nationwide	2000–2025	[117]

**Table 2:** Summary of key zoonotic pathogens at the animal-human interface in Cambodia, including host species, sample sizes, prevalence, location, study period, and primary data sources.

Disease/pathogen	Host(s)		Sample size	Key prevalence/findings	Location	Year(s)	Primary citation(s)
Melioidosis (*Burkholderia pseudomallei*)	Hospital patients		Case series	Severe sepsis and pneumonia; paediatric cases; high mortality; underdiagnosis linked to limited laboratory capacity; strong climatic associations	Provincial hospitals (Siem Reap, Phnom Penh, Takeo)	2008–2024	[130,131,133–138]
*Rickettsia* spp.	Ticks, fleas		Molecular survey	Multiple *Rickettsia* spp. detected in ectoparasites; confirms environmental circulation	Southern Cambodia	2024	[80]
	Traveller (case report)		1 confirmed case	*Rickettsia sibirica* infection in international traveller; confirms pathogenic spotted fever group circulation	Exposure in Cambodia	2024	[79]
Brucellosis (*Brucella spp.*)	Cattle, swine		Abattoir survey	Low seropositivity detected; comparable to Lao findings; limited national surveillance	Phnom Penh Province	2019–2020	[91,177]
Q Fever (*Coxiella burnetii*)	Cattle, goats		Abattoir survey	Seropositivity detected; spatial risk mapping performed	Phnom Penh & selected provinces	2019–2023	[91]
Hepatitis E Virus (HEV)	Swine		Molecular detection	Genotype 1 detected in swine; genotype 3 detected in river water; multiple genotypes circulating	Nationwide; river systems	2006–2009	[36,37]
	General population		Community survey	18.4% anti-HEV IgG; genotype 3 & 4 detected	Siem Reap	2015	[38]
	Pregnant women		1,565 women	11.6% seropositive	Siem Reap	2024	[39]
	Blood donors/ clinical cohorts		Multiple studies	Historically high exposure; declining IgG prevalence (1996–2017)	Phnom Penh	1996–2017	[40–42]
Japanese Encephalitis Virus (JEV)	Pigs		505 pigs (8 provinces)	65–74% seroprevalence; high infection rates in pigs >6 months; continuous peri-urban transmission	Multi-province	2006–2010	[66–69,72,178]
	Children (meningoencephalitis surveillance)		586 children	19% JE-positive; majority ≤12 years; major paediatric CNS disease	6 hospitals	2006–2008	[60]
	Vaccination programme		National data	Vaccination cost-effective; high campaign coverage; favourable safety profile	Nationwide	2010s	[63–65]
Influenza A viruses (H5, H7, H9, etc.)	Ducks, chickens		Live bird market surveillance	Ducks 51.3%, chickens 39.6% positive; co-infections detected; reassortment events; H9N2 endemic; H5N1 resurgence 2023–2025	Live bird markets; nationwide	2009–2025	[99,100,102–107,179]
	Market workers; confirmed cases		Occupational sampling; case clusters	Air samplers PCR-positive during peak circulation; ≤1% community seroprevalence; severe H5N1 clusters	Nationwide	2005–2025	[102,105–107]
*Trichinella* spp.	Pigs		242 pigs (4 provinces)	2.5% seropositive	Northeastern Cambodia	2021	[16]
	Outbreak cases		≥33 cases (8 deaths)	*Trichinella papuae* outbreak linked to raw wild pig meat	Kampong Thom	2017	[21]
*Taenia* spp.	Pigs		242 pigs (4 provinces)	11.2% seropositive for Taenia cysticerci; associated with free-roaming pigs and access to human faeces	Northeastern Cambodia	2021	[16]
	Community & molecular studies		Multiple provincial surveys	*T. saginata*, *T. solium* detected; hybrid *T. saginata–T. asiatica* reported; focal endemicity	Northern & coastal provinces	2011–2021	[14,15,17]
*Opisthorchis viverrini*	Snails (*Bithynia* spp.), fish		Field surveys	Confirmed in intermediate hosts; focal fish metacercarial infection	Kampong Cham; multi-province	2009–2017	[26–28]
	Community surveys		Multi-province studies	High endemicity in northern provinces; adult worm expulsion confirms active infection	Preah Vihear; Stung Treng	2011–2023	[29,30]
Visceral Leishmaniasis (*Leishmania* spp.)	Paediatric case		1 case	First reported VL case; required liver and bone marrow biopsy; diagnostic challenges	Cambodia	2022	[83]
Rabies virus	Dogs, humans	Not specified	High incidence relative to population; dog-mediated transmission; large unvaccinated dog population; close human–animal contact	Nationwide	2000–2025	[116]	

**Table 3:** Zoonotic pathogens at the livestock–human interface in Laos and Cambodia: key findings and structural drivers.

Pathogen group	Major transmission drivers	Key knowledge gaps	Priority one health opportunities
*Taenia* spp.	Free-roaming pigs, poor sanitation, raw pork consumption	Limited disease estimates, diagnostic limitations	Pig management, sanitation improvements, targeted surveillance
*Trichinella* spp.	Raw pork/wild pig consumption, limited meat inspection	Wildlife reservoir contribution poorly defined	Food safety education, meat inspection, wildlife surveillance
*Opisthorchis viverrini*	Raw fish consumption, aquatic transmission cycle	Reinfection dynamics, behavioural determinants	Community education, sanitation, integrated fish/snail surveillance
HEV	Pig reservoirs, informal slaughter, foodborne transmission	True human incidence poorly characterised	Pig surveillance, food safety interventions, community serosurveys
JEV	Pig amplification, mosquito vectors	Local ecological drivers of transmission	Pig sentinel surveillance, vaccination, vector monitoring
Rickettsial infections	Vector exposure, livestock-associated environments	Reservoir ecology, spatial risk mapping	Livestock surveillance, vector surveillance, clinician awareness
Brucellosis/Q fever	Livestock contact, reproductive fluids	Amount of human disease	Livestock surveillance, occupational health interventions
Influenza A	Live bird markets, poultry trade	Drivers of recent H5N1 resurgence	LBM surveillance, genomic surveillance, cross-border coordination
Rabies	Dog-mediated transmission	True mortality	Dog vaccination, integrated human-animal surveillance
AMR	Antimicrobial use in livestock and humans	Transmission pathways between sectors	Integrated AMR surveillance, stewardship programmes

AMR, Antimicrobial resistance; JEV, Japanese Encephalitis virus; HEV, Hepatitis E virus; LBM, Live bird market.

More recent work has extended these findings by showing that *T. solium* risk is spatially heterogeneous and firmly structured by environmental, demographic, and husbandry factors, with both spatial autocorrelation methods and multicriteria decision analysis identifying persistent high-risk foci in northern Laos [10,11]. A matched case-control study further identified consumption of raw and fermented pork, particularly from wild pigs, as the principal risk factor for *T. solium* taeniasis, highlighting an under-recognised transmission pathway linked to neurocysticercosis in the region [7]. Concurrently, scoping review and diagnostic evaluation studies have demonstrated substantial limitations in the sensitivity and specificity of both microscopic and molecular methods for detecting taeniasis and neurocysticercosis in low- and middle-income settings. This underscores the likelihood of systematic under-ascertainment and misclassification of infection presence [12,13].

Smallholder pig systems in northern Cambodia face similar sanitation and husbandry risks, though data on prevalence are limited. Molecular studies of *Taenia* tapeworms highlight important One Health issues at the human-animal-environment interface, comparable yet distinct from those in Laos. Early work used mitochondrial (*cox1*) gene sequencing and multiplex PCR to identify human *Taenia* infections, confirming *T. saginata* and *T. solium* among residents via faecal egg analysis, indicating zoonotic transmission linked to livestock and food practices [14]. Molecular diagnostics in residents detected definitive *T. saginata* infections [15], emphasising the need for genetic tools for accurate species identification in areas where morphology falls short. A survey of 242 pigs from 139 smallholder households in four provinces showed an ELISA seroprevalence of 11.2% for porcine *Taenia* cysticerci, with the highest prevalence in Preah Vihear, Cambodia [16]. Recently, the first hybrid of *T. saginata* and *T. asiatica* was found in Preah Vihear via sequencing of *cox1* and nuclear markers, pointing to mixed transmission and potential hybrid zoonoses in regions bordering Laos and Southeast Asia, where genetic interchange occurs [17].

These findings show that *Taenia* transmission in Cambodia involves complex host-parasite interactions, affecting public health, veterinary control, and food safety. Similar challenges occur in Laos, but with unique species and hybridisation that may influence control strategies. In both Laos and Cambodia, pigs eating human faeces and free-roaming management were significantly linked to cysticercosis [6,8], highlighting how husbandry and sanitation influence ongoing transmission risks linking animal reservoirs to potential human infection. Effective parasite control requires strategies that consider broader ecology, human behaviour, and diagnostic limitations, not just *T. solium*. This aligns with WHO priorities for controlling zoonotic helminthiases and explains slow progress in areas with extensive pig farming, poor sanitation, and limited diagnostics [2].

#### *Trichinella* spp.

The mapping of *Trichinella* spp. has further expanded understanding of the parasitic risk profile in Laos, where cases of trichinellosis were documented in the late 2000s [18,19]. In 2014, a cross-sectional study in four northern provinces demonstrated substantial human exposure to *Trichinella* spp., with a seroprevalence of 19.1% (95% CI 17.1-21.1) among 1,419 individuals. In parallel, examination of 728 pigs identified *Trichinella* larvae in 2.1% of animals, with most isolates identified as *T. spiralis*. These endemic patterns are consistent with outbreak investigations, including the 2005 Oudomxay outbreak in northwestern Laos, which estimated approximately 650 human cases, and highlight the persistence of foodborne transmission linked to the consumption of raw or fermented pork [20]. These geographic areas lacked both meat inspection infrastructure and routine veterinary care, reinforcing the link between zoonotic risk and structural health inequities.

In Cambodia, a documented outbreak of trichinellosis caused by *T. papuae* in Kampong Thom Province in 2017 resulted in at least 33 human infections and eight deaths following consumption of raw wild pig meat, with molecular diagnostics (PCR and sequencing) confirming *T. papuae* as the causative agent and highlighting links between wildlife, cultural practices, and human disease risk [21]. Complementary evidence from cross-sectional surveys in four rural Cambodian provinces found low but notable seroprevalence of *Trichinella* spp. exposure (2.5% in pigs) [16], indicating that smallholder pig production systems remain reservoirs for zoonotic transmission and reflecting risk factors such as feeding pigs food waste and poor management practices. These findings indicate that transmission is influenced by interactions among wildlife reservoirs, smallholder pig production systems, food preparation practices, and veterinary oversight.

#### Opisthorchis viverrini.

*O. viverrini* is a medically important food-borne liver fluke requiring *Bithynia* snails and freshwater cyprinid fish as intermediate hosts. *O. viverrini* is the dominant liver fluke in Laos and Cambodia and is strongly associated with cholangiocarcinoma; a closely related “sister” species, *Clonorchis sinensis*, causes identical disease processes but is distributed primarily in East Asia [22]. *O. viverrini* is highly endemic in Laos, particularly in lowland provinces along the Mekong River, where infection prevalence in some communities has historically exceeded 50% [23,24]. Transmission occurs through the consumption of raw or undercooked cyprinid fish containing metacercariae, a practice embedded in traditional dishes such as *koi pla* and related preparations. Chronic infection is strongly associated with hepatobiliary disease, including cholangitis and periportal fibrosis, and is a well-established risk factor for cholangiocarcinoma, a major public health concern in the region [24,25]. Despite periodic mass drug administration with praziquantel, reinfection remains common due to persistent culinary practices, inadequate sanitation, and ongoing environmental contamination, underscoring the need for integrated control strategies that combine chemotherapy, health education, improved sanitation, and food safety interventions [23,25].

*O. viverrini* is also an endemic and emerging parasitic infection in Cambodia. *O. viverrini* was confirmed in the first intermediate host, *Bithynia* snails, in Kampong Cham Province in 2017 through combined morphological and molecular identification, demonstrating active transmission potential in local aquatic environments [26]. Subsequent field research highlighted that *O. viverrini* infection has been reported in most Cambodian provinces, with a highly focal distribution and evidence of under-reported transmission, suggesting that locations from Preah Vihear to Takeo can have village-level prevalences >20% [27–29]. More recently, high endemicity of *O. viverrini* has been documented in northern Cambodia (Preah Vihear and Stung Treng provinces) by detecting eggs in faecal samples and confirming infection through adult worm expulsion, emphasising a significant human health problem in riparian areas [27,30]. These findings indicate that human behaviour, notably the consumption of raw or undercooked fish or fish products, local ecological conditions, and the parasite’s life cycle all contribute to sustained transmission, supporting the need for integrated One Health approaches to control opisthorchiasis in Laos and Cambodia.

#### Soil-borne helminths.

Complementary investigations identified domestic dogs as important reservoirs of intestinal helminths, including zoonotic species such as *Echinococcus granulosus*, further complicating control efforts [31]. These findings cast doubt on the long-term effectiveness of mass drug administration (MDA) strategies that target only human hosts, illustrating how multi-host parasite life cycles can undermine single-sector public health approaches. The presence of multiple definitive hosts, combined with environmental contamination and poor sanitation, created a highly permissive environment for sustained transmission. Despite extensive MDA programmes, little is known regarding zoonotic transmission of hookworms, *Strongyloides* spp., and other soil-transmitted helminths between humans and animals in Laos and Cambodia. Few One Health studies have investigated cross-species transmission pathways. This lack of evidence represents an important One Health research gap in both countries.

#### Hepatitis E virus.

Hepatitis E virus (HEV) is endemic in Laos and Cambodia and remains an important zoonotic pathogen linked to livestock production systems, particularly pig farming. Pigs serve as key reservoirs, and foodborne transmission, primarily through informal slaughter and consumption of undercooked pork, contributes to human infection. In backyard and smallholder systems characterised by limited food safety oversight and poor sanitation, HEV transmission reflects the same structural vulnerabilities that sustain parasitic and bacterial zoonoses.

In Laos, ACIAR-funded serological surveys across 16 provinces demonstrated high levels of HEV exposure in pigs, with seroprevalence of 51.2% (300/586) in abattoir pigs and 15.3% (46/301) in village pigs, indicating substantial circulation in both commercial and smallholder systems [32]. In a separate study, molecular screening of 181 young pigs in the northern provinces of Luang Prabang, Oudomxay, Xiengkhouang, and Houaphanh detected HEV RNA in 11.6% of the pigs, and 43.5% of village herds had at least one infected pig. Phylogenetic analysis classified Lao HEV strains as genotype 4, clustering with Chinese human and pig isolates, supporting a zoonotic transmission link between pigs and humans [33]. More recently, a cross-sectional serosurvey of 2,412 people across five Lao provinces (Central, Vientiane Capital; the North, Oudomxay and Luang Prabang; and the South, Champasak and Savannakhet) found 57.9% positive for anti-HEV IgG. Seropositivity increased with age and varied by province, indicating widespread exposure and marked geographic heterogeneity in transmission [34]. However, despite widespread HEV circulation in pigs and relatively high prevalence, hospital-based studies in Vientiane reported low HEV hospital inpatient incidence in humans [35], suggesting underdiagnosis or poor capture of infections in rural populations and highlighting the need for broader community-based serosurveys.

HEV is endemic in Cambodia, with domestic pigs serving as important reservoirs for zoonotic genotypes, and foodborne transmission through undercooked or contaminated pork representing a major route of human infection. HEV genotype 3 has also been identified in river water [36]. Although HEV genotype 1 RNA has also been reported in Cambodian pigs [37], this genotype is generally considered human-restricted, and the epidemiological significance of its detection in animals remains unclear, with no evidence to date supporting zoonotic transmission. In Siem Reap, 18.4% of the general population had anti-HEV IgG, and HEV genotypes 3 and 4 were detected [38], indicating active zoonotic circulation. Additionally, 11.6% of 1565 pregnant women in Siem Reap tested seropositive [39]. Sequential studies in Phnom Penh revealed high historical anti-HEV IgG prevalence that declined over two decades [40–42]. Among blood donors, 18.4% were anti-HEV IgG-positive, but HEV was detected in fewer than 1%, indicating that past infections are common but current viraemia is rare [42]. In patients with mild liver enzyme elevation, unexplained fever, or HIV, IgG seroprevalence exceeded 30%, while acute HEV infections were rare. A long-term study showed HEV IgG prevalence dropped from about 39% in 1996 to 9% in 2017, indicating reduced but ongoing transmission [40–42]. Together, the evidence from both countries underscores sustained, widespread HEV circulation across commercial and smallholder systems, driven by livestock reservoirs, foodborne and environmental transmission pathways, and structural vulnerabilities such as limited food safety oversight and traditional pork consumption practices that facilitate zoonotic transmission.

#### Neorickettsia sennetsu.

*N. sennetsu* is an under-recognised cause of febrile illness in mainland Southeast Asia, particularly in Laos and, to a lesser extent, Cambodia. Early work in Laos demonstrated relatively high exposure, with seroprevalence of anti-*N. sennetsu* antibodies reaching ~17% among blood donors and patients, alongside molecular confirmation of infection in both humans and freshwater fish, supporting a likely fish-borne transmission pathway associated with raw fish consumption [43]. Subsequent studies have reinforced that *sennetsu* neorickettsiosis may be a “neglected” infection contributing to undifferentiated fever in the region [44]. In Cambodia, evidence is more limited but indicates lower-level exposure; for example, serological studies in febrile populations report seroprevalence around 5–6%, suggesting sporadic but present transmission [45]. Despite this, clinical recognition remains poor, and the disease is rarely diagnosed, with very few reported cases globally, highlighting a substantial gap between exposure and confirmed disease in both Laos and Cambodia [44].

### Water-associated zoonoses

#### Schistosoma mekongi.

*S. mekongi* remains a geographically restricted but clinically significant parasitic infection along the Mekong River in southern Laos and northern Cambodia. Transmission is closely linked to the distribution of the intermediate host snail*, Neotricula aperta*, and to human water-contact activities such as fishing, bathing, and washing. During the 1980s and 1990s, very high prevalence levels were reported in endemic villages, often exceeding 50%, with substantial morbidity including hepatosplenic disease, portal hypertension, and growth retardation in children [46–48]. In Laos, endemic foci are concentrated in the Khong District of Champasak Province [46,49,50], while in Cambodia, transmission persists primarily in the provinces of Kratié and Stung Treng [46,51]. Over the past two decades, national control programmes based on repeated mass drug administration with praziquantel, combined with health education and improved surveillance, have markedly reduced prevalence and infection intensity in both countries [51,52]. However, complete interruption of transmission remains challenging due to persistent low-level transmission, ecological constraints related to the snail host, and evidence of zoonotic reservoirs (e.g., dogs and pigs), particularly in remote riverine communities [49,53].

### Vector-borne zoonoses

#### Japanese encephalitis virus.

Japanese encephalitis virus (JEV) is a leading cause of viral central nervous system infection in Laos and Cambodia. In Laos, JEV has been identified through prospective surveillance at Mahosot Hospital as one of the most common causes of encephalitis in both children and adults. Hospital-based and seroepidemiological studies have demonstrated substantial transmission, particularly in rural settings, precipitating the introduction and expansion of national vaccination programmes [54–59]. In Cambodia, two years of sentinel surveillance (2006–2008) across six Cambodian hospitals identified Japanese encephalitis (JE) in 19% (110/586) of children presenting with meningoencephalitis, with site-specific proportions ranging from 13% to 35%, and 95% of confirmed cases occurred in children ≤12 years of age [60]. The disease is also recognised in travellers to both Laos [61] and Cambodia [62].

JEV vaccination has been implemented in both Laos and Cambodia. In Cambodia, cost-effectiveness analyses conducted prior to vaccine introduction demonstrated that JEV vaccination would be highly cost-effective, given the substantial level of paediatric encephalitis and the long-term neurological sequelae that immunisation could prevent [63]. Subsequent mass vaccination campaigns using the live, attenuated SA14-14-2 JE vaccine achieved high community coverage in provinces such as Battambang, with operational lessons improving uptake and delivery efficiency [64], while post-campaign safety evaluations confirmed a favourable adverse event profile and no unexpected safety concerns [65]. Together, these studies demonstrate that JEV vaccination in Cambodia is economically justified, operationally feasible, safe, and effective in reducing the risk of severe childhood disease.

JEV transmission in Laos and Cambodia is closely linked to pig farming [66–72], rural landscapes, and ecological seasons. Pigs serve as amplifying hosts, maintaining viral circulation and increasing spillover risk during periods of high mosquito activity. A serological survey in northern Laos (May 2008-January 2009) showed a 74.7% JEV seroprevalence by hemagglutination inhibition (HI) serology, with 2.3% IgM positivity, indicating ongoing and recent transmission. These findings highlight intense, seasonal JEV activity in pigs, emphasising their role and the importance of livestock surveillance for human risk [71,73]. Transmission peaks at the start of the monsoon, when vector numbers, environmental contamination, and human-animal contact rise. Detecting JEV antibodies in pigs serves as an early warning for human outbreaks, aiding vaccination efforts and public health messaging.

In Cambodia, pigs are key to JEV transmission, maintaining viral circulation at the human-animal interface in rural and peri-urban areas. Serological testing of 505 pigs across eight provinces showed widespread JEV infection, with 65.7% and 63.5% seropositivity by HI and ELISA, respectively, and a high infection rate (95.2%) in pigs over six months old [72]. Sentinel surveillance near Phnom Penh revealed high, continuous JEV transmission with rapid seroconversion, indicating frequent exposure [67]. Comparative studies confirmed high transmission in both rural and peri-urban sites, though seasonal dynamics vary with ecological context and vector abundance [68]. Pigs develop high viraemia, making them the principal amplifying hosts, but models suggest that pig-to-pig transmission contributes minimally to persistence, with vector transmission dominating [69]. Entomological studies support this, showing mosquito vectors prefer pig feeding, reinforcing their role as transmission bridges [70]. Serological studies in domestic birds suggest they also play a role as enzootic hosts [74]. High seroprevalence in small farms and limited farmer awareness highlight pigs’ role in maintaining transmission and the need for integrated One Health strategies [66]. These findings emphasise the importance of livestock-based surveillance for seasonal zoonotic risk and demonstrate how land-use and agricultural practices influence transmission dynamics.

#### *Rickettsia* and *Orientia* spp.

Rickettsioses are emerging zoonotic, vector-borne diseases in Southeast Asia caused by intracellular *Rickettsia* and *Orientia* spp. bacteria that pose growing threats to public health, animal welfare, and food security. Human rickettsial infections, particularly scrub typhus and murine typhus, are well-documented causes of febrile illness in Laos. Early prospective studies demonstrated that *Orientia tsutsugamushi* (the causative agent of scrub typhus) and *Rickettsia typhi* (the causative agent of murine typhus) accounted for a substantial proportion of acute undifferentiated fever among patients presenting to Mahosot Hospital in Vientiane, establishing rickettsioses as major but previously under-recognised contributors to febrile disease in the country [75–77]. Animals have been used as sentinels and infection proxies to identify rickettsial “hotspots”. Nationwide abattoir-based serology conducted between January 2022 and April 2023 across 18 provinces tested 821 animal samples for antibodies against scrub typhus, typhus, and spotted fever group rickettsiae. Thirty-two samples (3.9%) were seropositive overall, comprising 25 cattle (9.9%) and seven pigs (2.4%), with no positives detected in buffalo. Multivariable analysis identified breed and age as significant predictors of seropositivity (*p* < 0.05). Spatial-temporal analysis revealed a significant cattle cluster in southern Laos (*p* = 0.0056), indicating focal environmental exposure and supporting the role of livestock surveillance in mapping rickettsial risk [78].

Recent evidence highlights the growing recognition of rickettsial infections as environmentally mediated zoonoses in Cambodia, reinforcing their relevance within a One Health framework. A case of *R. sibirica* infection was reported in an international traveller returning from Cambodia, confirming the presence of pathogenic spotted fever group rickettsiae in the country and demonstrating the potential for cross-border dissemination through human mobility [79]. Complementing this, molecular surveillance of ectoparasites collected from southern Cambodian provinces detected multiple *Rickettsia* species in ticks and fleas, providing direct evidence of pathogen circulation within arthropod vectors at the human-animal interface [80]. Together, these findings indicate that rickettsial transmission in Cambodia is sustained through vector–animal–human ecological networks, likely influenced by livestock contact, wildlife reservoirs, land-use patterns, and limited vector surveillance. They underscore the importance of integrating entomological monitoring, clinical awareness, and cross-border surveillance to address emerging rickettsial risks in Southeast Asia.

#### Leishmania.

Visceral leishmaniasis (VL), increasingly reported in Southeast Asia but not previously documented in Laos, was investigated through PCR screening of 1,015 people living with HIV, serological testing of 511 febrile patients, and 159 ruminants [81]. While all HIV samples were PCR-negative, low-level seropositivity was detected in one human case (0.4%) and in 3.1% of ruminants, suggesting possible undetected circulation of *Leishmania* in Laos and highlighting the need for further epidemiological investigation and increased clinical awareness. Building on this surveillance, a multi-year survey of phlebotomine sandflies across seven provinces in Laos revealed high species diversity, particularly in karstic cave environments, including a new species and previously unreported taxa. Although no *Leishmania* was detected, the identification of an unknown *Trypanosoma* species highlights the likely presence of unrecognised sandfly-borne pathogens and the need for expanded surveillance [82]. The first reported case of VL in Cambodia involved a child from Stung Trung, presenting with fever, anaemia, thrombocytopenia, and splenomegaly, features that overlap with many infectious and haematologic conditions [83]. Diagnosis required invasive investigations, including multiple bone marrow biopsies and a liver biopsy to demonstrate intracellular *Leishmania* spp., highlighting significant diagnostic challenges and the likelihood of under-recognition of VL in Cambodia. To date, no confirmed autochthonous cases of cutaneous leishmaniasis have been reported in Laos or Cambodia.

### Direct animal contact and market interface zoonoses

#### Brucellosis (*Brucella* spp.).

Brucellosis has long been under-recognised within national zoonoses and neglected tropical disease control agendas in Southeast Asia, despite its well-documented public health and economic impact in other regions. In livestock-keeping populations, *Brucella* spp. infections are associated with reproductive losses, reduced productivity, and chronic economic hardship, while in humans, infection typically presents as a non-specific febrile illness that is frequently misdiagnosed as typhoid, dengue, or leptospirosis [84]. Human brucellosis has rarely been reported in Laos or Cambodia, and despite occasional detection of *Brucella* exposure in livestock, confirmed human cases remain largely undocumented, suggesting that the disease may be either uncommon or substantially underdiagnosed due to limited diagnostic capacity and low clinical awareness. The absence of routine diagnostic testing and structured surveillance has reinforced a low index of suspicion among health professionals, perpetuating neglect across both veterinary and human health sectors. Human infection typically occurs through contact with infected birth products, aborted foetuses, reproductive secretions, or consumption of unpasteurised dairy products. Consumption of unpasteurised dairy products is less common in Laos and Cambodia than in many endemic regions.

In the animal sector, this knowledge gap began to be addressed in Laos through an ACIAR-funded biosurveillance project in 2006 [85], followed by a more extensive DTRA-funded programme initiated in 2015 and running through to 2025, which generated the first national-level data on brucellosis in livestock using abattoir-based serology [86–89]. Seropositivity was detected across multiple species, with particularly notable findings in smallholder goats, a species often excluded from routine surveillance despite its importance in peri-urban and upland production systems [86,90]. Abattoir-based surveillance indicated a very low apparent seroprevalence of brucellosis in large ruminants in Laos. In a 2019 survey across six provinces, goats showed slightly higher but still low seropositivity in targeted studies (approximately 1.4%). These findings place Laos at the lower end of the spectrum when compared with international settings, where baseline prevalence in livestock has ranged from 1.4% in cattle to as high as 20% in goats, and where economic analyses show vaccination-only strategies can yield benefit-cost ratios up to 21.3, while test-and-slaughter approaches are frequently not cost-effective [86,88].

In Cambodia, a cross-sectional serosurvey of 540 goats sampled in 2019–2020 from six provinces in southern Cambodia (Phnom Penh, Kandal, Takeo, Kampong Speu, Kampot, and Preah Sihanouk) estimated the true seroprevalence of antibodies to *Brucella* spp. at only 0.1% (95% CI 0.0–1.0), and no significant risk factors were identified, suggesting that brucellosis was rare in the goat populations during the period of the study [91]. To date, evidence for *B. suis* circulation in pigs in Laos and Cambodia remains limited, and published data are insufficient to define its epidemiological importance.

#### Streptococcus suis.

*S. suis* is an important zoonotic pathogen across mainland Southeast Asia. In Vietnam, it is a leading cause of adult bacterial meningitis and sepsis [92,93]. Human infection is typically associated with occupational exposure to pigs or pork products and with the consumption of raw or undercooked pork and fresh pig blood dishes (such as *Larb Dip* in Laos), practices that remain culturally embedded in parts of the region. Outbreaks have been reported in northern Vietnam and northern Thailand [92,94], with the most common *S. suis* serotypes being 2, 4, 5, 9, 14, 16, 24, and 31 [95]. Serotype 2 is the most frequently identified cause of invasive human disease in Cambodia [95,96], although the serotype causing disease in Laos has not yet been identified [95]. Limited studies have been performed in Laos using PCR on CNS disease patient samples with *S. suis* detected at relatively low levels [97,98]; however, recent studies indicate that the disease may be more prevalent than originally thought. The level of disease is likely underestimated due to limited diagnostic capacity and under-recognition, highlighting the need for strengthened laboratory surveillance, food safety interventions, and risk communication strategies across the GMS [93–95]. Although foodborne transmission through raw pork and pig blood consumption is important, *S. suis* is included in this section because occupational exposure to pigs and pork products remains a major risk factor.

#### Influenza A virus.

Surveillance in Cambodia and Laos shows that avian influenza A viruses persistently circulate in poultry, especially in live bird markets (LBMs) [99–101]. Particular concern centres on avian-origin influenza A viruses such as H5, H7 and H9 because of their zoonotic potential and capacity for pandemic emergence. A longitudinal study in Cambodia found influenza A in 51.3% of ducks and 39.6% of chickens, with frequent co-infections (H5 and H9) [99]. LBM workers face high inhalation risks, with viral RNA detected in all personal air samples collected during periods of high virus circulation and viable virus detected in half of these samples [102]. In Laos (2009–2010), active poultry surveillance revealed multiple introductions of H5N1, including clades 2.3.4.1, 2.3.4.2, and 2.3.2.1, showing repeated cross-border incursions rather than a single endemic strain [101]. In Cambodia, low pathogenic A(H9N2) viruses have circulated endemically in poultry since at least 2013, with molecular analyses demonstrating sustained local evolution and reassortment within the BJ/94-like h9-4.2.5 sub-lineage. These viruses formed multiple genotypes and are closely related to strains in Vietnam, indicating ongoing cross-border movement through the regional poultry trade. Studies of highly pathogenic A(H5N1) clade 2.3.2.1c viruses during 2014–2016 showed significant genetic diversity and reassortment, highlighting the co-circulation of multiple avian influenza lineages in domestic poultry [103]. Beyond H5 and H9, sporadic detections of unusual or emerging viruses, including H7N3 associated with duck mortality [104] and H14N2 in ducks [105], emphasise the dynamic ecology of avian influenza in the region. These findings underscore the need for sustained surveillance across the GMS to detect reassortment, cross-border introductions, and new subtypes. Deep sequencing of H5N1 from humans and ducks revealed mainly low-frequency intra-host variants shaped by purifying selection and drift, as well as recurring human-adaptive mutations (e.g., PB2 E627K, HA A150V, Q238L) during natural spillover, indicating ongoing opportunities for adaptation despite short infection durations [106].

Human infection remains relatively uncommon but epidemiologically significant. Community serological studies indicate a seroprevalence of ≤1%, suggesting that while spillover events occur, widespread subclinical transmission has been limited. Nevertheless, Cambodia has experienced multiple clusters of severe human H5N1 disease since 2005, with a marked resurgence of confirmed cases from late 2023 through 2024–2025, prompting intensified surveillance and genomic investigations [107,108]. The resurgence of human H5N1 infections in Cambodia since 2023 contrasts with many regions experiencing widespread poultry outbreaks but relatively few severe human cases. The reasons for this apparent discrepancy remain unclear and warrant further investigation.

#### Coronaviruses.

Bat-borne coronaviruses are widely distributed across Asia, with their diversity and transmission dynamics shaped by ecological and environmental factors, including land-use change and habitat overlap between wildlife and humans [109]. Longitudinal surveillance in Cambodia has demonstrated increased circulation of alpha- and beta-coronaviruses among juvenile and immature bats, suggesting that host age structure plays an important role in viral amplification and maintenance within bat populations [110]. Phylogenetic and phylogeographic analyses consistently identify horseshoe bats (*Rhinolophus* spp.) as key reservoirs of sarbecoviruses, with SARS-CoV and SARS-CoV-2 exhibiting mosaic genomic ancestry derived from viruses circulating in bat populations across southern China and mainland Southeast Asia [111–113]. Regional studies from Laos and Cambodia further highlight substantial genetic diversity of bat coronaviruses, underscoring the breadth of the viral reservoir in this region [114]. Recent immunological investigations have identified geographically variable humoral reactivity to both pandemic and bat-derived coronavirus variants, which may reflect prior exposure to related viruses, although such findings remain indirect and do not establish a direct progenitor of SARS-CoV-2 in humans [115].

#### Rabies.

Rabies remains endemic in both Laos and Cambodia, where it constitutes a substantial but under-recognised cause of preventable mortality, driven predominantly by domestic dog-mediated transmission. Estimates from Cambodia suggest a high annual incidence of human rabies deaths relative to population size, with transmission sustained by large, largely unvaccinated dog populations and close human–animal contact [116]. In Laos, although fewer data are available, rabies is similarly considered endemic, with under-reporting compounded by limited diagnostic capacity and fragmented surveillance systems [117]. Consistent with broader analyses of pathogen discovery and surveillance capacity, rabies is strongly influenced by health system strengthening, laboratory infrastructure, and reporting mechanisms, suggesting that current estimates likely underestimate the true incidence [117]. Strengthening integrated One Health approaches, including mass dog vaccination, improved surveillance, and expanded access to post-exposure prophylaxis (PEP), remains critical to reducing rabies transmission and achieving regional elimination targets [118].

Rabies virus in Southeast Asia exhibits notable genetic diversity, reflecting long-term endemic circulation and regional viral evolution. Studies from Laos, Thailand, Vietnam, and Cambodia consistently demonstrate the presence of multiple co-circulating lineages within the broader Asian clade, often structured into distinct phylogenetic subclades [119–121]. For example, analyses in Laos and Cambodia have identified at least two divergent lineages or subclades circulating simultaneously, suggesting sustained local transmission cycles with limited cross-border homogenisation [119–121]. In Thailand, genetic characterisation of the glycoprotein gene indicates relatively conserved but still distinguishable variants among dog populations, consistent with localised evolution [122]. More recent work in Vietnam has further highlighted this diversity, identifying four genetically distinct groups, including the SEA1 and SEA3 subclades, underscoring the complex phylogeographic patterns in the region [123].

### Environmental zoonoses associated with animal raising systems

#### Q fever (*Coxiella burnetii*).

Q fever in humans, known as coxiellosis in animals, is caused by *Coxiella burnetii* and represents a neglected bacterial zoonosis in Southeast Asia, despite well-established occupational and environmental transmission pathways [124]. In livestock, *C. burnetii* infection is associated with reproductive disorders and reduced productivity [124]. In contrast, in humans, it causes acute and chronic febrile illnesses that are rarely diagnosed due to limited clinical awareness and diagnostic capacity [124].

In Laos, multiple studies have shown *C. burnetii* seropositivity in livestock. The earliest, from 2006, sampled cattle and buffalo across five provinces, revealing significant differences between provinces (*p* < 0.001) and high seroprevalence in Xayaboury (15.9%) and lower rates in Luang Prabang (3.8%), Houaphanh (1.3%), and Phongsaly (0.6%) [85]. A 2015 study found a 1.2% overall seroprevalence in buffalo, cattle, pigs, and goats across several provinces, with a cluster of seropositive cattle in Xayaboury bordering Thailand [90]. A 2019 pilot study detected 5.7% seropositivity in Xiengkhouang cattle and buffalo from six provinces [88]. A 2020 study of 4,247 samples from 18 abattoirs showed higher prevalence in Oudomxay (buffalo 5.3%, cattle 2.8%), Xayaboury (cattle 5.0%), and Luang Namtha (cattle 4.8%) [89]. A 2016–2017 serosurvey of 1,458 goats across five provinces showed a 4.1% seroprevalence [86], with higher risk linked to province, breed, and age.

In Cambodia, a cross-sectional serosurvey of 540 goats sampled in 2019–2020 from six provinces in southern Cambodia (Phnom Penh, Kandal, Takeo, Kampong Speu, Kampot, and Preah Sihanouk) identified an overall *C. burnetii* seroprevalence of 7.2% (95% CI 5.3–9.7), indicating active circulation of Q fever within smallholder goat production systems [91]. Seropositivity was significantly associated with sex and commune, with female goats having nearly ten times higher odds of seropositivity than males (OR 9.7, 95% CI 2.7–35.5), suggesting localised transmission and highlighting the potential for human exposure to Q fever in communities where goats are raised in close proximity to households [91].

Recent serological studies in Cambodia have revealed *C. burnetii* exposure among febrile patients. Among 321 with persistent fever, 4.3% were seropositive, indicating prior exposure [125]. A separate investigation of 54 patients found 22.2% had antibodies, suggesting Q fever might cause some undiagnosed febrile illnesses, despite no PCR-confirmed cases [126]. Although Q fever has not been confirmed in humans in Laos (but see Syhavong et al), livestock surveillance has detected *C. burnetii* in multiple species and provinces, indicating that the pathogen persists in animals. Consistent antibody detection and aerosol transmission suggest human exposure may occur but go unnoticed due to limited testing and awareness, making Q fever an under-recognised cause of febrile illness, as recently observed in Cambodia.

#### Melioidosis.

Human melioidosis is highly endemic in Laos, with the causative agent *Burkholderia pseudomallei*. The disease is rarely zoonotic, with transmission primarily through exposure to contaminated soil and water. Limited surveillance, weak diagnostic capacity, and low awareness have constrained case detection, particularly in animals, raising concern that the true level remains underestimated and may increase further as diabetes prevalence rises in this predominantly agricultural population [127–129]. A 16-year study in Laos and Cambodia demonstrated that climatic factors such as humidity, rainfall, and wind were predictive of cases, highlighting environmental transmission [130].

In Laos, retrospective serological analyses of febrile and sepsis patients suggest broader exposure than routine surveillance captures [131], indicating that clinical case counts likely underestimate the true presence, a pattern also observed in hospital-based studies.

An abattoir-based serosurvey of 917 livestock across Laos demonstrated substantial exposure to *B. pseudomallei*, with a significant spatial cluster detected along the southwest border in Savannakhet Province (*p* = 0.041). Overall, seroprevalence varied by species, with the highest in cattle (22.8%), followed by buffalo (19.7%) and pigs (4.0%). Temporal analysis showed a marked seasonal peak, with the highest seroprevalence observed in January 2020 (17.0%). The geographic overlap between livestock seropositivity and known human case areas supports the use of livestock as sentinels for environmental risk and highlights widespread, under-recognised exposure in agricultural settings [132]. Clinical case series from Cambodia have documented culture-confirmed melioidosis presenting as severe sepsis and pneumonia, with substantial mortality in both adults and children, often linked to delayed diagnosis and limited microbiological capacity [133–135]. Paediatric emergence in Siem Reap further highlighted the vulnerability of children exposed to contaminated environments [136], while prospective pulmonary studies confirmed melioidosis as an under-recognised cause of severe respiratory infection [137].

The dangers of a lack of awareness of *B. pseudomallei* infection include the risk of exposure to veterinary and para-veterinary staff during livestock post-mortems. The importance of increasing awareness through capacity-building initiatives, such as the DTRA-funded Cambodia Training Event for Awareness of Melioidosis (C-TEAM), emphasises that underdiagnosis remains a major barrier to control. [138]. Together, the evidence from both countries shows that melioidosis persists where environmental reservoirs, occupational soil exposure, seasonal climatic amplification, and limited diagnostic infrastructure converge.

#### Anthrax.

Anthrax, caused by *Bacillus anthracis*, is an endemic but under-reported zoonotic disease in both Laos and Cambodia, primarily affecting rural, livestock-dependent communities. In neighbouring Vietnam [139–143] and Thailand [144–148], there are numerous reports of anthrax infections in humans and animals. In Laos, sporadic outbreaks most commonly occur in the southern provinces in cattle and buffalo, with human cases typically linked to the slaughtering or handling of infected animals and consumption of contaminated meat. Several outbreaks have been reported in provinces such as Salavan, where there is a high level of awareness, often involving cutaneous anthrax but occasionally gastrointestinal disease [149,150]. Across both countries, limited diagnostic capacity, under-recognition, and weak surveillance systems likely contribute to substantial underestimation of true incidence.

### Antimicrobial resistance at the human–animal interface

Antimicrobial resistance compounds the neglect of bacterial zoonoses by undermining empirical treatment strategies in settings with limited diagnostics. Antimicrobial resistance (AMR) poses a growing challenge in Laos, Cambodia, and other LMICs in the GMS, complicating efforts to control neglected bacterial zoonoses. In livestock production systems in LMICs, where unregulated antimicrobials are widely used for disease prevention and growth promotion, selective pressure contributes to the emergence and spread of resistant organisms at the animal–human interface [4].

A national review by Chansamouth and colleagues [151] highlighted rising extended-spectrum beta-lactamase (ESBL)-producing *Escherichia coli* in clinical isolates, the emergence of carbapenem resistance, high inpatient antimicrobial use, and widespread antibiotic self-medication, while noting major surveillance gaps in the animal sector. Farm-level investigations further revealed widespread, sometimes prophylactic, antibiotic use, with heterogeneous knowledge and practices among farmers [152]. Cross-border molecular studies confirmed the presence and plasmid-mediated transfer of resistance determinants between animals, meat products, and humans along the Thailand-Laos border, reinforcing AMR as a regional One Health issue [153].

In Laos, collaborative studies between the Lao-Oxford-Mahosot Hospital-Wellcome Trust Research Unit (LOMWRU) and DLF have made important contributions toward filling this evidence gap, particularly regarding antimicrobial use in livestock systems and associated resistance profiles. Initial research conducted in the mid-2000s, based primarily on clinical isolates from Mahosot Hospital, suggested that AMR prevalence in Laos was relatively low compared to neighbouring countries, likely due to limited access to and use of antimicrobials at the time [154]. However, this situation has evolved considerably over the past decade, with growing access to veterinary pharmaceuticals, increased intensification of livestock production, and insufficient regulation of antimicrobial use in both human and animal health sectors. More recent surveillance studies, integrating clinical samples from humans and animal samples from abattoirs, have begun to detect significant signals of emerging resistance. Of particular concern was the identification of colistin-resistant *E. coli* in pigs, reported in a 2024 study leveraging samples collected through the DTRA-funded abattoir-based surveillance program [155]. Subsequent livestock surveillance demonstrated high levels of multidrug resistance in *E. coli* and *Salmonella* from pigs and chickens, including resistance to critically important antimicrobials such as third-generation cephalosporins, fluoroquinolones, and colistin [156].

In Cambodia, AMR is being driven by overlapping human, animal, and environmental factors that reflect the broader One Health context of infectious disease transmission. Widespread access to antibiotics without prescription, informal livestock production systems, and limited diagnostic microbiology capacity contribute to substantial selective pressure for resistant pathogens. Research led by the Cambodia-Oxford Medical Research Unit (COMRU), based at Angkor Hospital for Children in Siem Reap, has been central to characterising the epidemiology of AMR in Cambodia through clinical microbiology surveillance, genomic studies of bacterial pathogens, and investigation of antimicrobial prescribing practices. COMRU has established diagnostic laboratory capacity, contributed to national AMR surveillance systems and antimicrobial stewardship initiatives, and generated data on key resistant pathogens, including *E. coli*, *Klebsiella pneumoniae*, and *Staphylococcus aureus,* in Cambodian hospitals [157]. These studies are embedded within wider regional initiatives, including the ACORN clinical AMR surveillance network [158–160], which integrates clinical and microbiological data to improve understanding of infection syndromes, antimicrobial use, and treatment outcomes across low- and middle-income settings. Together, this work highlights how AMR in Cambodia is shaped by interconnected drivers, including antibiotic misuse across the human and veterinary sectors, limitations in healthcare infrastructure, and livestock-associated transmission pathways, underscoring the need for integrated One Health surveillance and stewardship strategies.

The intersection of AMR with zoonotic NTDs highlights the need to strengthen antimicrobial stewardship within a One Health framework. Efforts to control neglected zoonotic diseases must therefore incorporate responsible antimicrobial use in livestock, improved diagnostic capacity, and coordinated surveillance across human and animal health sectors. Without addressing AMR, progress toward effective management of neglected bacterial zoonoses is likely to remain fragile and uneven.

### Drivers of zoonotic disease risk in Laos and Cambodia

#### Socio-cultural behaviours in the One Health context.

Understanding zoonotic transmission requires attention not only to biological pathways but also to the socio-cultural and economic contexts that shape everyday interactions between humans, animals, and their environments. While biomedical tools, such as vaccines, diagnostics, and treatment protocols, are central to disease control, their effectiveness is often undermined by entrenched practices, behavioural norms, and structural barriers. In Laos, traditional pig production systems remain largely extensive, low-input, and household-managed, with pigs frequently allowed to roam freely, fed swill, and slaughtered informally. There is also the community risk profile, in which women and children care for pigs and chickens and are therefore more at risk of zoonotic diseases these livestock carry. These practices are deeply embedded in rural livelihoods, shaped by cultural norms, labour dynamics, and subsistence economies. Combined with limited access to veterinary services, poor cold-chain infrastructure, and low prioritisation of animal health at the household level, these conditions sustain the transmission cycles of key zoonotic pathogens [161].

A complementary dimension of this challenge was revealed through a longitudinal serological study of wet market vendors, conducted in partnership with LOMWRU. Over multiple sampling rounds, vendors were found to have elevated and fluctuating seropositivity against *O. tsutsugamushi*, *R. typhi*, and *Leptospira* spp., pathogens associated with rodent exposure, contaminated environments, and proximity to animals [162]. These findings confirmed that wet markets function as high-risk occupational settings, where zoonotic exposure is both routine and cumulative. Markets are not only hubs for trade and consumption but also social and economic institutions, where regulatory enforcement is often weak, and health risks are poorly understood or tolerated as part of daily life.

**Fig 2:**
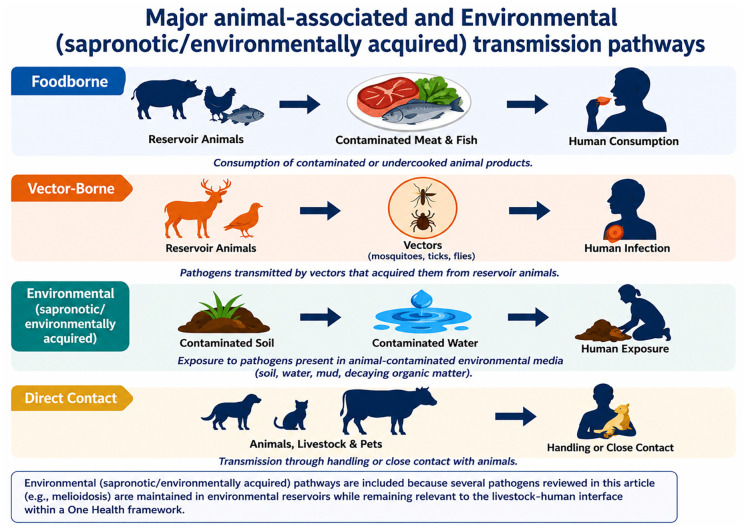
Major animal-associated and environmentally acquired (sapronotic) transmission pathways relevant to pathogens reviewed in this article. Illustration created by the authors using OpenAI ChatGPT image-generation tools (Version 1.2026.049).

**Fig 3:**
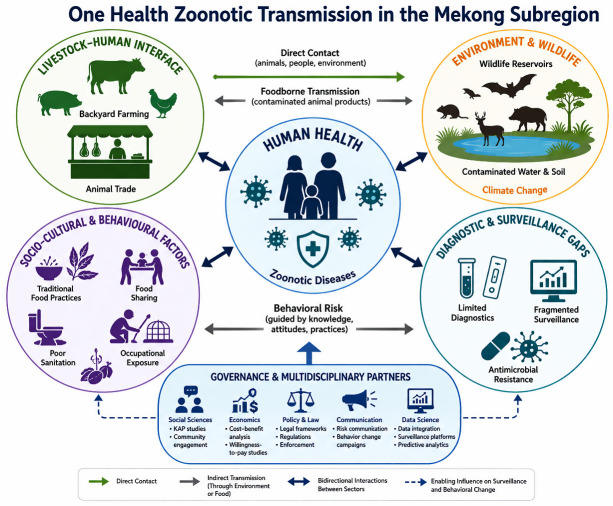
One Health framework illustrating interactions among livestock-human interfaces, wildlife reservoirs, environmental change, socio-cultural drivers, governance and multidisciplinary partners, and diagnostic and surveillance systems influencing zoonotic disease transmission. The figure was generated using OpenAI ChatGPT image-generation tools (Version 1.2026.119) and subsequently edited by the authors.

Together, these findings emphasise the importance of adopting a One Health perspective that considers transmission pathways (Fig 2), as well as environmental, socio-economic and behavioural factors. They also point to the need for integrated surveillance approaches that include occupational risk groups, such as market vendors, butchers, and livestock handlers, who are often overlooked in formal health systems. Addressing zoonotic risk in such contexts requires cross-sectoral collaboration, participatory research, and policy tools that reflect the complex interplay between behaviour, livelihood, and disease ecology (Fig 3).

#### Climate change.

Climate change is increasingly recognised as an important driver of global zoonotic disease dynamics [4], including in Laos and Cambodia. Changes in temperature, rainfall patterns, and the increasing frequency of extreme weather events influence pathogen survival, vector ecology, and host–pathogen interactions, thereby altering the spatial and temporal distribution of zoonotic infections. Vector-borne diseases are particularly sensitive to climatic variability, as warmer temperatures and altered precipitation regimes can expand vector habitats and prolong transmission seasons. In the GMS, climatic conditions influence the transmission of pathogens such as JEV [163] and rickettsial [164,165] species. In contrast, environmental pathogens such as *B. pseudomallei*, the causative agent of melioidosis, show strong associations with rainfall and soil moisture [166,167]. These patterns suggest that climate variability may amplify existing zoonotic transmission pathways in rural agricultural systems where human-livestock-environment interactions are already intense.

In Laos and Cambodia, climate-sensitive zoonoses occur within landscapes undergoing rapid ecological and agricultural change. Seasonal monsoon dynamics, flooding, and shifts in land use affect mosquito breeding habitats, wildlife distribution, and livestock production systems, thereby influencing opportunities for pathogen spillover [168–170]. For example, the incidence of melioidosis has been shown to correlate with humidity and rainfall across Laos and Cambodia, highlighting the role of environmental exposure in agricultural communities [130]. Such climate-linked ecological drivers underscore the need for integrated One Health surveillance systems that monitor environmental, animal, and human health indicators simultaneously.

#### Trade and regional animal movement.

Regional trade networks also play an important role in shaping zoonotic disease risk in the GMS. Rapid expansion of cross-border livestock and poultry trade, particularly along corridors linking Laos and Cambodia with China and Vietnam, has increased the movement of animals, animal products, and associated pathogens. China represents a major market for livestock and agricultural commodities from mainland Southeast Asia, and the intensification of regional trade has been associated with the cross-border spread of infectious diseases affecting both humans and animals [171–173]. Informal trade routes and limited veterinary oversight further increase the risk that zoonotic pathogens may move across national boundaries undetected.

The expansion of regional infrastructure and trade corridors, including those associated with the Belt and Road Initiative, has accelerated human and animal movement and market integration both globally and in the GMS [174,175]. While these developments contribute to economic growth, they also increase opportunities for pathogen dissemination through livestock transport, live animal markets, and complex supply chains linking rural production systems to urban markets. These dynamics highlight the importance of coordinated transboundary surveillance and One Health collaboration between countries in the region to detect and mitigate zoonotic disease threats associated with increasing regional trade [171–173].

## Discussion

This review shows that multiple zoonotic pathogens remain entrenched at the livestock-human interface in Laos and Cambodia despite more than two decades of investments in surveillance, research, behaviour change and good practice. Across these pathogen groups, a consistent pattern emerges: zoonotic diseases remain entrenched not because they are poorly understood biologically, but because the structural conditions that sustain transmission remain largely unchanged.

The persistence of zoonotic diseases in Laos and Cambodia reflects the interaction of three reinforcing drivers. First, livestock production systems that sustain rural livelihoods also create persistent opportunities for zoonotic transmission. Second, many zoonotic infections remain diagnostically invisible due to limited laboratory capacity and fragmented surveillance systems, meaning that their true impact is rarely captured by routine health reporting. Third, cultural food practices and occupational exposures at the livestock–human interface are deeply embedded within local economies and social systems, making behavioural change alone insufficient to interrupt transmission.

The first driver of persistence is the structure of livestock production systems that dominate rural economies in the GMS. A central finding is the persistence of zoonotic pathogens within smallholder livestock production systems. Backyard pig and poultry production, informal slaughter practices, and the proximity of animals to households create sustained opportunities for pathogen transmission. These production systems are deeply embedded within rural livelihoods and food systems, meaning that zoonotic disease control cannot rely solely on biomedical interventions. These intervantions must also address the economic and social realities of livestock keeping. Evidence from Laos and Cambodia demonstrates that interventions targeting individual pathogens often fail to achieve sustained impact when underlying husbandry practices, food preparation behaviours, and sanitation conditions remain unchanged.

The second driver is the skewed nature of diagnostics and surveillance. Many zoonotic diseases identified in this review circulate primarily within livestock populations or environmental reservoirs before spilling over into humans. However, surveillance systems in both countries remain heavily weighted toward human clinical detection, which is understandable given the limited resources available. As a result, zoonotic pathogens frequently remain undetected until they cause severe disease in people. Livestock-based surveillance programmes, particularly abattoir-based sampling systems implemented in Laos, demonstrate the potential of integrated monitoring platforms to detect multiple pathogens and antimicrobial resistance signals simultaneously. Expanding such surveillance frameworks across the region could provide earlier warning of emerging zoonotic threats while strengthening routine monitoring of endemic diseases.

Diagnostic capacity represents another critical constraint. Many zoonotic infections described in this review present as non-specific febrile or neurological illnesses that overlap clinically with more commonly recognised diseases such as dengue, malaria, or typhoid fever. In rural healthcare settings where laboratory diagnostics are limited, clinicians often rely on syndromic management, resulting in substantial underdiagnosis of zoonotic infections. Experience from Laos has demonstrated that strengthening microbiological and research capacity can substantially increase pathogen detection, underscoring how surveillance intensity and diagnostic capability, rather than true emergence alone, strongly influence observed patterns of infectious disease discovery [176]. This diagnostic invisibility contributes directly to policy neglect, as diseases that are not routinely detected are unlikely to be prioritised within national health programmes. Strengthening microbiological and molecular diagnostic capacity in provincial laboratories, combined with improved clinician awareness of zoonotic diseases, will therefore be essential for improving disease recognition and surveillance.

The third driver relates to socio-cultural and behavioural practices embedded within rural livelihoods. Practices such as consuming raw or undercooked meat and fish, informal slaughter, and close daily contact with livestock are integral components of rural livelihoods and food traditions in both Laos and Cambodia. These practices are often resistant to change through conventional public health messaging alone. Effective interventions, therefore, require participatory approaches that engage communities in risk communication and incorporate culturally appropriate strategies for behavioural change. Evidence from wet-market surveillance and occupational studies highlights that certain population groups, including vendors, butchers, and livestock handlers, experience repeated and cumulative exposure to zoonotic pathogens and should therefore be prioritised within surveillance and prevention programmes. Successful One Health implementation requires multidisciplinary collaboration extending beyond clinicians and veterinarians. Social scientists can identify behavioural drivers through knowledge, attitudes and practices studies; agricultural economists can evaluate the feasibility and cost-effectiveness of interventions; legal and policy experts can strengthen governance and regulatory frameworks; communication specialists can improve risk communication and community engagement; and data scientists can support integrated surveillance systems, data sharing, and predictive analytics.

Environmental and ecological factors influence the spread of zoonotic diseases across the region. Climate variability, monsoon patterns, and land use affect vectors, wildlife reservoirs, and pathogens. Climate change may worsen transmission by changing conditions that support pathogen survival and vectors. Incorporating environmental monitoring into One Health systems could enhance early detection of seasonal or climate-related disease risks.

The inclusion of AMR within this review highlights an additional dimension of the livestock–human interface. Increasing antimicrobial use in livestock production systems, combined with expanding access to antibiotics in both human and veterinary sectors, has created conditions conducive to the emergence and spread of resistant pathogens. Evidence from Laos and Cambodia demonstrates that resistance determinants can be transmitted between animals, food products, and humans, underscoring the need for coordinated antimicrobial stewardship policies. Addressing AMR within a One Health framework will be critical to maintaining the effectiveness of treatments for both zoonotic and non-zoonotic infections.

Taken together, the evidence presented in this review suggests that effective control of zoonotic diseases in the GMS requires a shift away from pathogen-specific responses toward integrated One Health systems. Such systems should combine coordinated surveillance across human, animal, and environmental sectors with strengthened diagnostic capacity, community-engaged behavioural interventions, and improved regulation of livestock production and food safety. Regional collaboration will also be essential, as porous borders and expanding livestock trade networks facilitate the movement of pathogens across national boundaries.

Several successful examples already exist within the GMS. Abattoir-based surveillance programmes in Laos have demonstrated the value of integrated livestock monitoring for multiple zoonotic pathogens and antimicrobial resistance. Pig-based surveillance for Japanese encephalitis virus has provided early warning of seasonal zoonotic risk, while longitudinal wet-market surveillance programmes have identified occupational exposure risks among vendors and animal handlers. Together, these initiatives demonstrate the feasibility and practical value of integrated One Health monitoring systems in resource-constrained settings and provide models that could be expanded across the region. Formal zoonotic disease prioritisation exercises such as the CDC One Health Zoonotic Disease Prioritization (OHZDP) process and WOAH prioritisation frameworks could help Laos and Cambodia align surveillance investments with national risk profiles and optimise allocation of limited resources.

The principal knowledge gaps and One Health intervention opportunities identified in this review are summarised in Table 3. Strengthening provincial diagnostic capacity, expanding integrated surveillance platforms, and prioritising behavioural interventions targeting raw meat consumption, informal slaughter, and wet-market exposures represent practical opportunities for improving zoonotic disease control in Laos and Cambodia.

Despite substantial advances in research and surveillance over the past two decades, zoonotic diseases continue to impose significant health and economic stress in Laos and Cambodia. Addressing these challenges will require sustained investment in One Health infrastructure, stronger cross-sectoral governance, and policies that recognise the complex interactions between livestock production, environmental change, and human health. By confronting the structural drivers of zoonotic disease persistence rather than focusing solely on individual pathogens, countries in the GMS can develop more effective and equitable strategies to manage zoonotic disease risks. Together, these findings suggest that the persistence of zoonotic diseases in the region reflects structural characteristics of livestock production systems, food cultures, and health system capacity rather than simply gaps in scientific knowledge. Effective control requires multidisciplinary One Health approaches integrating human and animal health professionals with social scientists, economists, environmental scientists, legal experts, communication specialists and data scientists (Box 1).

**Box 1. Key messages for zoonotic NTD control at the livestock–human interface**

- Zoonotic neglected tropical diseases (NTDs) remain endemic at the livestock–human interface in Laos and Cambodia despite two decades of surveillance evidence.
- Persistent transmission reflects fragmented One Health governance, limited diagnostic capacity, informal livestock production systems, and entrenched food and husbandry practices.
- Many infections, including *Taenia solium*, trichinellosis, brucellosis, Q fever, rickettsioses, melioidosis, hepatitis E virus, and Japanese encephalitis virus, are substantially underdiagnosed and underreported.
- Livestock-based surveillance, particularly abattoir systems, provides a scalable platform for integrated detection of zoonotic pathogens and antimicrobial resistance.
- Effective control requires multidisciplinary One Health approaches integrating surveillance, behavioural change, veterinary and public health systems, together with expertise from the social sciences, economics, law, communication and data science.
- Addressing zoonotic NTDs is not only a public health priority but also an imperative for poverty reduction and agricultural development in the Greater Mekong Subregion.

## Abbreviations

ACIAR: Australian Centre for International Agricultural Research
ACORN: A Clinically Oriented Antimicrobial Resistance Surveillance Network
AMR: Antimicrobial Resistance
BJ/94: Beijing/1994 lineage (influenza virus lineage)
CDC: Centers for Disease Control and Prevention
CNS: Central Nervous System
COMRU: Cambodia-Oxford Medical Research Unit
DALY: Disability-Adjusted Life Year
DLF: Department of Livestock and Fisheries (Lao PDR)
DTRA: Defense Threat Reduction Agency
ELISA: Enzyme-Linked Immunosorbent Assay
ESBL: Extended-Spectrum Beta-Lactamase
FAO: Food and Agriculture Organization of the United Nations
GMS: Greater Mekong Subregion
HEV: Hepatitis E Virus
HI: Haemagglutination Inhibition
HIV: Human Immunodeficiency Virus
JEV: Japanese Encephalitis Virus
LBM: Live Bird Market
LMIC: Low- and Middle-Income Country
LOMWRU: Lao-Oxford-Mahosot Hospital-Wellcome Trust Research Unit
MDA: Mass Drug Administration
MORU: Mahidol Oxford Tropical Medicine Research Unit
NTD: Neglected Tropical Disease
OHHLEP: One Health High-Level Expert Panel
OHZDP: One Health Zoonotic Disease Prioritization
OR: Odds Ratio
PCR: Polymerase Chain Reaction
PEP: Post-Exposure Prophylaxis
QGIS: Quantum Geographic Information System
RNA: Ribonucleic Acid
SARS-CoV: Severe Acute Respiratory Syndrome Coronavirus
SARS-CoV-2: Severe Acute Respiratory Syndrome Coronavirus 2
STH: Soil-Transmitted Helminth
VL: Visceral Leishmaniasis
WHO: World Health Organization
WOAH: World Organisation for Animal Health
